# Supplementary material for: Patterns of Intron Gain and Loss in Fungi
Source: PLoS Biol. 2004 Nov 30;2(12):e422. doi: 10.1371/journal.pbio.0020422 (PMC532390; doi:10.1371/journal.pbio.0020422)
Supplement: Table S1 — Also available at http://genes.mit.edu/NielsenEtAl/. (4.3 MB ZIP). [file pbio.0020422.st001.zip › NielsenEtAl/html/195.html]

AN4080.1.NCU00389.1.MG05730.1.FG07241.1


```
 CLUSTAL W (1.82) Multiple Sequence Alignments - Introns Inserted


Sequence 1: MG05730.1	363 aa
Sequence 2: FG07241.1	364 aa
Sequence 3: NCU00389.1	357 aa
Sequence 4: AN4080.1	364 aa
Alignment Length: 379 aa
Number Identitical Residues: 280 aa
Alignment Score (without introns) 11342


MG05730.1 	-------~-------~MAKTQKNKATSYHLG~QLKAKLAKLKRELLTPSSGGGGGG-A~G
NCU00389.1	MSTTVDK0IKEIEAE0MARTQKNKATSYHIG~QLKAKLAKLKRELLTPSGGGGGGGGA~G
FG07241.1 	-------~-------~MAKTQKNKATSFHLG~QLKAKLAKLKRELLTPSGGGGGGG-T~G
AN4080.1  	-------~-------~MARTQKNKNTSYHLG1QLKAKLAKLKRELLTPSGGGGGGSGA1G
          	                **:***** **:*:* *****************.*****..: *

MG05730.1 	FDVARTGVASI~GFIGFPSVGKSTLMSRLTGQHSEA~AAYEFTTLTSVPGQVIYNG1APL
NCU00389.1	FDVARTGVASI~GFIGFPSVGKSTLMSRLTGQHSEA~AAYEFTTLTSVPGQVTYNG1APL
FG07241.1 	FDVARTGVASI~GFIGFPSVGKSTLMSRLTGQHSEA~AAYEFTTLTSVPGQVVYNG1APL
AN4080.1  	FDVARTGVASV2GFIGFPSVGKSTLMSRLTGQHSEA1AAYEFTTLTTVPGQVLYNG~AKI
          	**********: ************************ *********:***** *** * :

MG05730.1 	QIIDLPGIIEGAKDGRGRGRQVIAVAKTCHLIFIVLDVNKPLTDKRVIESELEGFGIRIN
NCU00389.1	QIIDLPGIIEGAKDGRGRGRQVIAVAKTCHLIFIVLDVNKPLTDKRVIEAELEGFGIRIN
FG07241.1 	QIIDLPGIIEGAKDGRGRGRQVIAVAKTCHLIFIVLDVNKPLTDKRVIEAELEGFGIRIN
AN4080.1  	QILDLPGIIQGAKDGKGRGRQVIAVAKTCHLIFIVLDVNKPLVDKKVIENELEGFGIRIN
          	**:******:*****:**************************.**:*** **********

MG05730.1 	KSPPNITFKKKEKGGLNITSTVPLTHIDHD~EIKAVMSEYRINSADIAIRCDATIDDLID
NCU00389.1	KEPPNITFKKKDKGGLNITSTVPLTHIDND~EIRAVMSEYKISSADIAIRCDATIDDLID
FG07241.1 	KEPPNITFRKKDKGGLNVTSTVPLTHIDHG~EIKAVMSEYRINSADITIRCDATVDDLID
AN4080.1  	KQPPNIMFKKKDKGGISITSTVPLTHIDND0EIKAVMSEYKISSADISIRCDATIDDLID
          	*.**** *:**:***:.:**********:. **:******:*.****:******:*****

MG05730.1 	ILEAKSRS~YIPVIYVLNKIDAISIEELDLLYRIPNAVPISSEHGWNIDELMEAM2WEKL
NCU00389.1	VLEAKSRS~YIPVIYVLNKIDAISIEELDLLYRIPNSVPISSEHGWNIDELMEAM~WEKL
FG07241.1 	VLEAKSRS2YIPVVYCLNKIDSISIEELDLLYRIPNAVPISSEHGWNIDELMEAM~WDKL
AN4080.1  	VLEAKSRA~YIPVVYALNKIDAITIEELDLLYRIPNAVPISSEHGWNIDELLEMM~WEKL
          	:******: ****:* *****:*:************:**************:* * *:**

MG05730.1 	NLVRVYTKPKGKLPDYSAPVVLRSNRCTVEDF0CNAIHRSIAEVFKTAIVYGKSVKHQPQ
NCU00389.1	KLVRVYTKPKGKMPDYSAPVVLRSNRCTVEDF0-----------FKVAIVYGKSVKHQPQ
FG07241.1 	SLVRVYTKPKGKQPDYSQPVVLRATRCTVEDF0CNAIHRSITEVFKTAIVYGKSVKHQPQ
AN4080.1  	NLRRIYTKPKGKAPDYTAPVVLRANACTVEDF0CNAIHRTIKDQFKQAIVYGRSVKHQPQ
          	.* *:******* ***: *****:. ****** ..:   :  . ** *****:*******

MG05730.1 	RVGLAHELADEDIG1CIAPGWVQWQCVAVE-
NCU00389.1	RVGLSHELADEDIV1TIVKR-----------
FG07241.1 	RVGLSHELCDEDVG~RLSRNTRNQLWTPVAA
AN4080.1  	RVGLTHELADEDIG~SRPFCEAYGTIGLTV-
          	****:***.***:
```
